# Dimerization Drives Proper Folding of Human Alanine:Glyoxylate Aminotransferase But Is Dispensable for Peroxisomal Targeting

**DOI:** 10.3390/jpm11040273

**Published:** 2021-04-06

**Authors:** Mirco Dindo, Giulia Ambrosini, Elisa Oppici, Angel L. Pey, Peter J. O’Toole, Joanne L. Marrison, Ian E. G. Morrison, Elena Butturini, Silvia Grottelli, Claudio Costantini, Barbara Cellini

**Affiliations:** 1 Department of Medicine and Surgery, University of Perugia, 06132 Perugia, Italy; mirco.dindo@oist.jp (M.D.); silvia.grottelli@unipg.it (S.G.); costacla76@gmail.com (C.C.); 2 Department of Neurosciences, Biomedicine and Movement Sciences, University of Verona, 37134 Verona, Italy; giulia.ambrosini@univr.it (G.A.); elisa.oppici@gmail.com (E.O.); elena.butturini@univr.it (E.B.); 3 Departamento de Química Física, Unidad de Excelencia de Química Aplicada a Biomedicina y Medioambiente e Instituto de Biotecnología, Facultad de Ciencias, Universidad de Granada, 18071 Granada, Spain; angelpey@ugr.es; 4 Bioscience Technology Facility, Department of Biology, University of York, York YO23 3GE, UK; peter.otoole@york.ac.uk (P.J.O.); joanne.marrison@york.ac.uk (J.L.M.); ian.morrison@york.ac.uk (I.E.G.M.)

**Keywords:** alanine:glyoxylate aminotransferase, pyridoxal phosphate, peroxisome, peroxisomal import, dimerization, protein folding, fluorescence recovery after photobleaching

## Abstract

Peroxisomal matrix proteins are transported into peroxisomes in a fully-folded state, but whether multimeric proteins are imported as monomers or oligomers is still disputed. Here, we used alanine:glyoxylate aminotransferase (AGT), a homodimeric pyridoxal 5′-phosphate (PLP)-dependent enzyme, whose deficit causes primary hyperoxaluria type I (PH1), as a model protein and compared the intracellular behavior and peroxisomal import of native dimeric and artificial monomeric forms. Monomerization strongly reduces AGT intracellular stability and increases its aggregation/degradation propensity. In addition, monomers are partly retained in the cytosol. To assess possible differences in import kinetics, we engineered AGT to allow binding of a membrane-permeable dye and followed its intracellular trafficking without interfering with its biochemical properties. By fluorescence recovery after photobleaching, we measured the import rate in live cells. Dimeric and monomeric AGT displayed a similar import rate, suggesting that the oligomeric state per se does not influence import kinetics. However, when dimerization is compromised, monomers are prone to misfolding events that can prevent peroxisomal import, a finding crucial to predicting the consequences of PH1-causing mutations that destabilize the dimer. Treatment with pyridoxine of cells expressing monomeric AGT promotes dimerization and folding, thus, demonstrating the chaperone role of PLP. Our data support a model in which dimerization represents a potential key checkpoint in the cytosol at the crossroad between misfolding and correct targeting, a possible general mechanism for other oligomeric peroxisomal proteins.

## 1. Introduction

About one third of all known proteins display an oligomeric structure, and most of them are homodimeric enzymes [1]. Genetic saving and the gain of catalytic activity in functionally-obligate dimers may account for the importance of dimerization, although structural advantages, including higher thermodynamic stability and protection against misfolding and aggregation, likely play a major role [1,2]. Protein engineering techniques to create artificial monomeric forms have been instrumental in understanding the impact of dimerization on purified proteins [1], but whether these results can be extended to the cellular environment in which the folding of the protein takes place has remained unexplored. Several factors other than the intrinsic physico-chemical properties of the polypeptide chain influence the energy landscape inside a cell, including not only the crowded environment, the vectorial synthesis, and the presence of molecular chaperones, but also the possibility to be imported in a subcellular organelle [3,4]. In the latter case, folding and targeting are strictly intertwined processes. Indeed, proteins destined for the endoplasmic reticulum or mitochondria must be extensively unfolded and acquire the tertiary and quaternary structure once imported [5,6]. On the contrary, proteins destined for the nucleus or peroxisomes are imported in a fully-folded state and dimerization/oligomerization may occur before or after the import [7]. While the monomer/dimer equilibrium has been shown to influence the import and the functional properties of nuclear proteins [8,9,10], the impact of the quaternary assembly on the import of peroxisomal proteins is still debated [7,11,12].

Peroxisomal matrix proteins are synthesized in the cytosol and post-translationally imported by specific peroxisomal targeting sequences (PTS) [11]. Most proteins contain a PTS type 1 (PTS1), a C-terminal tripeptide generally matching the consensus sequence [S/A/C]-[K/R/H]-[L-M], which interacts with the cytosolic Pex5p receptor [7,11,13]. The cargo-Pex5p complex forms the docking complex with Pex14p and Pex13p at the peroxisomal membrane, where the cargo protein translocates via a poorly understood mechanism. Some evidence supports an “oligomeric protein import model”, where homo- or hetero-oligomerization occurs before import [14,15,16,17,18,19], while others are in favor of a “monomeric import model”, where the binding of the Pex5p receptor blocks oligomerization until the protein is released in the peroxisomal matrix [16,20,21,22,23]. However, these models have been derived by using Green Fluorescent Protein-fusion proteins in intact cells, or in vitro import assays based on isolated peroxisomes or semi-intact cellular systems that fail to fully recapitulate the import kinetics of the native protein in the cellular environment.

To address this problem, we used, as model human peroxisomal alanine:glyoxylate aminotransferase (AGT), a dimeric pyridoxal 5′-phosphate (PLP)-dependent enzyme whose functional deficit causes primary hyperoxaluria type I (PH1) [24,25]. PH1 is a rare inherited disease characterized by the progressive accumulation of calcium oxalate kidney stones due to the oxidation of accumulating glyoxylate [26]. Human AGT is present as two polymorphic forms, named major and minor allele, the latter characterized by two amino acid changes, Pro11Leu and Ile340Met [27]. Although the Pro11Leu mutation generates a putative N-terminal mitochondrial targeting sequence, protein mistargeting is prevented unless PH1-linked mutations are present that destabilize the dimeric structure and enhance interaction with molecular chaperones [28,29,30,31]. To assess the contribution of dimerization to AGT folding and peroxisomal targeting, here, we compared the two native dimeric forms of the protein encoded by the major (D_Ma_) and the minor (D_Mi_) allele, with the corresponding artificial monomeric forms (M_Ma_ and M_Mi_) obtained by the interfacial mutations R118A/F238S/F240S. In a previous study, we characterized the M_Mi_ form, and we reported that it is catalytically inactive but folded and able to bind PLP [32]. Here, we found that forced monomerization increases protein aggregation and degradation but does not prevent peroxisomal import. By resorting to a modified form of the protein able to interact with a fluorescent probe in live cells, we determined the kinetics of peroxisomal import and found that import depends on proper folding and not on quaternary structure acquisition. These results illuminate on the cellular fate of AGT with potential implications for the pathogenesis of PH1 and give useful insights into the general principles at the basis of the import of other matrix peroxisomal proteins.

## 2. Materials and Methods

### 2.1. Materials

PLP, pyridoxine (PN), L-alanine, sodium glyoxylate, NADH, rabbit muscle L-lactic dehydrogenase (LDH), isopropyl-b-D-thiogalactopyranoside, MG132, chloroquine, and cycloheximide, were obtained from Sigma-Aldrich (St. Louis, MO, USA). The FlAsH-EDT2 probe was purchased from Thermo Scientific (Waltham, MA, USA). 8-anilino-1-naphthalenesulfonic acid (ANS) was purchased from Molecular Probes (Eugene, OR, USA). Ham’s F12 Glutamax medium without PN was purchased from Invitrogen (Carlsbad, CA, USA). Rabbit polyclonal anti-AGT human, rabbit polyclonal anti-Pro11, and guinea pig antiperoxisomal catalase antibodies were kindly provided by Prof. C.J. Danpure (University College London). Anti-rabbit, anti-guinea pig and anti-mouse horseradish peroxidase (HRP) antibodies were purchased from GE Healthcare (Chicago, IL, USA). Oligonucleotides for site-directed mutagenesis were purchased from Eurofins Genomics (Anzinger, Germany). All other chemicals were of the highest grade commercially available.

### 2.2. Site Directed Mutagenesis

The R118A, F238S, and F240S mutations were introduced on the available pAGT-Ma and pAGT-Mi constructs for bacterial expression [33], as well as on the pcDNA3.1AGT-Ma and pcDNA3.1AGT-Mi vectors for eukaryotic expression [34] using the Quick Change Mutagenesis Kit (Stratagene, San Diego, CA, USA) and the primers previously reported [32]. The sequence encoding for the CCPGCC motif able to specifically bind the FlAsH-EDT2 dye was introduced at position 762 into the AGT coding sequence of the pAGT-Ma and pcDNA3.1AGT-Ma vectors by insertional mutagenesis. The primers used were AGT-FlAsH1 (5′-CTTCTGGGGCTGTGACTGTTGTCCGGACCAGCCCAGGATG-3′ and its complement) and AGT-FlAsH2 (5′-CTGTGACTGTTGTCCAGGCTGTTGTGACCAGCCCAGGATG-3′ and its complement). Each construct was confirmed to be free of mutations by DNA sequencing. The obtained pAGT-FlAsH-Ma and pcDNA3.1-FlAsH-Ma were then subjected to site-directed mutagenesis to obtain constructs encoding the corresponding monomeric forms by introducing the R118A, F238S, and F240S mutations. The amino acid sequence of each species under study is reported in Figure S1.

### 2.3. Expression and Purification

*Escherichia coli* BL21(DE3) cells, transformed with vectors encoding D_Ma_, D_Mi_, M_Ma_, M_Mi_, D_Ma_-FlAsH, M_Ma_-FlAsH, were grown in 4.5 L of Luria broth at 37 °C to an absorbance at 600 nm of 0.4–0.6. Expression was induced with 0.1 mM isopropyl-b-D-thiogalactopyranoside for 15 h at 30 °C. Cells were harvested and resuspended in 20 mM sodium phosphate buffer, pH 7.4, containing 0.5 M NaCl, 20 mM imidazole, and 100 µM PLP. β-mercaptoethanol (5 mM) was added to culture of proteins containing the FlAsH motif. Lysozyme was added to a final concentration of 0.2 mg/mL, and the culture was incubated for 15 min at room temperature. After a freeze–thaw, the lysate was centrifuged at 30,000× *g* for 30 min at 4 °C. Then, the lysate was loaded on a HisPrep FF 16/10 equilibrated with 20 mM sodium phosphate buffer, pH 7.4, containing 0.5 M NaCl, 40 mM imidazole (plus 5 mM β-mercaptoethanol for proteins containing the FlAsH motif). A linear gradient was then applied (0–100% in 200 mL) with the same buffer containing 500 mM imidazole. The proteins eluted between 200 and 300 mM imidazole. After addition of 100 µM PLP, the protein solution was concentrated; imidazole and unbound coenzyme were removed by extensive washing with 100 mM potassium phosphate buffer (KP), pH 7.4 (plus 5 mM β-mercaptoethanol for proteins containing the FlAsH motif), using Amicon Ultra 10 concentrators (Amicon). The protein concentration of dimeric and monomeric species was determined using extinction coefficients at 280 nm of 9.54 × 10^4^ M^−1^ cm^−1^ and 4.61 × 10^4^ M^−1^ cm^−1^, respectively. The PLP content of each enzymatic form was determined by releasing the coenzyme in 0.1 M NaOH using the extinction coefficient of 6600 M^−1^ cm^−1^ at 388 nm. The apo forms of the FlAsH tagged proteins were prepared as previously reported adding 5 mM β-mercaptoethanol in the buffers [35].

### 2.4. Determination of the Enzyme-PLP Equilibrium Dissociation Constant (K_D(PLP)_) of M_Ma_, M_Mi_, D_Ma_-FlAsH, M_Ma_-FlAsH

The K_D(PLP)_ of D_Ma_-FlAsH was calculated by measuring the intrinsic fluorescence quenching (λexc 280 nm; λem 336 nm) of the apoenzyme (0.1 μM) incubated for 24 h in the presence of PLP at a concentration range of 0.01–10 μM. The K_D(PLP)_ of M_Ma_ and M_Ma_-FlAsH were determined by measuring the circular dichroism (CD) signal at 430 nm of 10 μM apoenzyme upon 24 h incubation in the presence of PLP at concentrations ranging from 1 to 300 μM. Data were fitted to the following quadratic equation based on the tight-binding hypothesis [36]:

(1) $$Y=Y_{\max}\;\frac{\left[E\right]t+\left[\mathrm{PLP}\right]t\;+K_{D\left(\mathrm{PLP}\right)}-\;\sqrt{{\left(\left[E\right]t+\left[\mathrm{PLP}\right]t\;+K_{D\left(\mathrm{PLP}\right)}\right)}^{2}-4\left[E\right]t\left[\mathrm{PLP}\right]t\;}}{2\left[E\right]t}$$

where [E]t and [PLP]t represent total concentrations of the mutant and PLP, respectively; Y refers to either intrinsic fluorescence quenching or 430 nm dichroic signal changes at each PLP concentration; and Y_max_ refers to the aforementioned changes when all enzyme molecules are complexed with the coenzyme.

### 2.5. Kinetic Parameters

The kinetic parameters for the overall transamination reaction of the alanine/glyoxylate pair were determined at 0.1 μM enzyme concentration in the presence of saturating PLP concentrations by varying the substrate concentration at a fixed saturating cosubstrate concentration in KP 0.1 M, pH 7.4, at 25 °C. The specific transaminase activity of cellular clones was determined by incubating each cellular lysate with L-alanine and glyoxylate at saturating concentrations in KP 0.1 M, pH 7.4, at 25 °C. In both cases, the reaction was stopped by adding trichloroacetic acid 10% (*v*/*v*), and pyruvate production was measured using the spectrophotometric assay coupled with LDH, as previously reported [37].

### 2.6. Size Exclusion Chromatography

Size exclusion chromatography analyses were performed on an Akta fast protein liquid chromatography system (GE Healthcare, Chicago, IL, USA) using a Superdex 200 10/300 GL column at room temperature. The mobile phase used for M_Ma_, M_Mi_, D_Ma_-FlAsH, and M_Ma_-FlAsH was KP 0.1 M, pH 7.4. Detection was set at 280 nm, the flow rate at 0.5 mL/min and the injection volume was 100 μL. Each sample was dissolved in KP 0.1 M, pH 7.4, at different concentrations and incubated overnight at 25 °C (a sufficient time to reach equilibrium between monomer and dimer). Monomer and dimer concentration were determined by integrating the area under the curve and expressed as percent of total area of all protein-related peaks, as previously described [32], using the Unicorn 5.01 (GE Healthcare) software. According to Manning et al. [38], the K_D(dim-mon)_ value was determined by fitting the data to the following equation:

(2) $$\%D=\frac{\left[\left(8\left[E\right]+K_{D\left(\dim-\mathrm{mon}\right)}\right)-K_{D\left(\dim-\mathrm{mon}\right)}^{2}+16{\;K}_{D\left(\dim-\mathrm{mon}\right)}{\left[E\right]}^{\frac{1}{2}}\right]}{0.08\left[E\right]}$$

where %D is the percentage of dimer to total enzyme and [E] represents the total enzyme concentration in dimer equivalents. A plot of log[%D/0.04(100-%D)^2^] with respect to log[E] yields a straight line of slope 1. When log[%D/0.04(%D-100)^2^] = 1, K_D(dim-mon)_ = [E].

### 2.7. Spectroscopic Measurements

Absorption measurements were made using a Jasco V-550 (Jasco-Europe, Cremella (LC), Italy) spectrophotometer with 1-cm path-length quartz cuvettes at a protein concentration of 1–10 μM in KP 0.1 M, pH 7.4. Near UV-visible and far-UV CD spectra and thermal denaturation curves were recorded on a Jasco J-710 spectropolarimeter (Jasco-Europe, Cremella (LC), Italy) equipped with a Peltier temperature-controlled compartment by using 1 cm and 1 mm path-length cells, respectively. The enzyme concentration was 0.1–15 μM. Routinely, three spectra were recorded at a scan speed of 50 nm/min^−1^ with a bandwidth of 2 nm and averaged automatically.

### 2.8. Differential Scanning Calorimetry

Experiments were performed in a capillary VP-DSC microcalorimeter with a cell volume of 135 µL. Buffer used was Na-Hepes 20 mM; NaCl 200 mM, pH 7.4; and 0.2 mM β-mercaptoethanol. Proteins in the holo-form were prepared in this buffer with 0.1 mM PLP. Scans were performed in a 2–110 °C range at scan rates from 1.5–4 °C/min. Data were corrected for the instrumental response time and analyzed using a two-state irreversible model with first order kinetics [39]. The validity of the model was confirmed by comparing data at different scan rates and protein concentrations as already described [39].

### 2.9. Isothermal Titration Calorimetry

Experiments were performed in a VP-ITC200 microcalorimeter with a cell volume of 205 µL. Experiments were carried out in Na-Hepes 20 mM, NaCl 200 mM, or KP 0.1 M at pH 7.4 in the presence of 0.2 mM β-mercaptoethanol. The PTS1 binding domain of Pex5p (Pex5p-pbd) was placed in the titration syringe at 290 µM, and AGT variants at 9–11 µM (in monomer) in the sample cell (occasionally, the cell was filled with buffer in blank titrations). Titrations were performed by an initial injection of 0.5 µL, followed by fifteen injections of 2.5 µL spaced 200 s. Titrations were carried out at 10 °C in the high feedback mode. Data were analyzed using the software provided by the manufacturer, using a single-type of sites binding model and fixing the stoichiometry to 1 due to low affinities.

### 2.10. Cell Culture, Transfection, and Lysis

Chinese hamster ovary cells stably expressing glycolate oxidase (CHO-GO) were cultured at 37 °C under O_2_/CO_2_ (19:1) atmosphere in Low-B6 medium consisting of a custom-made B6 free Ham’s F12 medium (Invitrogen, Carlsbad, CA, USA) supplemented with fetal bovine serum (10%, *v*/*v*), penicillin (100 units/mL), streptomycin (100 µg/mL), and zeocin (0.4 µg/mL). PN concentration in the Low-B6 medium was <<0.3 µM [40]. CHO-GO cells were transfected using the Turbofect™ Transfection Reagent (Fermentas, Waltham, MA, USA) according to the manufacturer’s instructions. In order to obtain stable clones, 24 h after transfection, the selection agent geneticin (G418, 1 mg/mL) was added to the medium of transfected cells. Cells were then kept in culture for at least two weeks to eliminate all the cells not expressing AGT. The homogeneous and stable expression of AGT of each clone was checked by Western blot and immunofluorescence microscopy.

Cell pellets were lysed in phosphate buffered saline (PBS), pH 7.2, plus protease inhibitor cocktail Complete Mini (Roche, Basilea, Switzerland), by five freeze/thaw cycles followed by treatment with DNAse (1 unit) at RT for 45 min. The latter procedure was able to disrupt organelles completely, thus, allowing peroxisomal and mitochondrial AGT to be extracted, as previously shown by our group and others [34,41]. The whole cell extract was separated by centrifugation (12,900× *g*, 10 min, 4 °C) to obtain the soluble fraction. The pellets were then resuspended in an equal volume of denaturing gel loading buffer to obtain the insoluble fraction. The protein concentration of the soluble cell lysate was determined in quadruplicate by using the Bradford protein assay.

### 2.11. Western-Blot and Pulse-Chase Experiments

5–10 micrograms of soluble and/or insoluble cell lysate were loaded per lane on a 10% SDS-PAGE gel along with the Precision plus protein KaleidoscopeTM (Bio-Rad, Hercules, CA, USA) or the SharpMass VI (Euroclone, Pero (MI), Italy) molecular mass markers. Proteins were then transferred on a nitrocellulose membrane using the iBlot device (Invitrogen), followed by blocking on 5% milk (*w*/*v*) in TBST buffer (10 mM Tris-HCl, pH 7.2; 0.1 M NaCl; and 1% Tween20) at 37 °C for one hour. For AGT detection, the membrane was incubated with the polyclonal rabbit anti-AGT primary antibody (dilution 1:6000), the anti-Leu11 primary antibody (dilution 1:2000), or the anti-Pro11 antibody (dilution 1:2000) for 15 h at 4 °C. Peroxidase-conjugated anti-rabbit IgG was used as secondary antibody (dilution 1:10,000). Indicated cells were treated with MG132 (10 µM) or chloroquine (50 µM) for 24 h before the immunoblot analysis. Band detection was performed by using the enhanced chemi-luminescence (ECL) substrate reagent using a Chemidoc system (Biorad). Band volume (intensity/mm^2^) was quantified using the software QuantityOne. To control for protein loading, protein quantification in the cellular lysates was performed in quadruplicate for each sample, blotted membranes were inspected by staining with Ponceau solution (Sigma-Aldrich, St. Louis, MO, USA), and a non-specific band responsive to the anti-AGT antibody also present in the negative control (non-transfected cells), was analyzed in each blot. All the blots presented in this work were consistent with a similar protein loading among each sample.

Cross-linking was performed using bis-N-succinimidyl-(pentaethylene glycol) ester (BS(PEG)_5_) (Pierce, Waltham, MA, USA). Twenty-five micrograms of the whole cellular lysate were cross-linked with BS(PEG)_5_ at 1 mM or 10 mM final concentrations and quenched in 0.5 M Tris-HCl, pH 7.5, after 30 min. Ten micrograms of each sample were analyzed by Western blotting.

In order to determine the half-life of each species, cells were treated with cycloheximide, a known inhibitor of ribosomal protein synthesis [42]. Briefly, cycloheximide (10 µg/mL) was added to the medium of cells at 70–80% confluence. At different times (0, 8, 12, and 24 h) cells were harvested, lysed, and AGT levels were measured by Western blot, as described above.

### 2.12. Immunofluorescence Microscopy (IFM)

AGT subcellular localization was investigated by confocal microscopy experiments performed both in fixed and live cells. In the first case, approximately 3 × 10^5^ cells were seeded into each well of a 24-well plate containing a 13-mm glass coverslip. Twenty-four hours later, cells were fixed in 4% (*w*/*v*) paraformaldehyde, permeabilized with 0.3% Triton X-100 in PBS, and blocked in 3% BSA in PBS. For the immunolabelling, rabbit polyclonal anti-human AGT and anti-peroxisomal catalase from guinea pig [43] were used as primary antibodies, and Alexa Fluor conjugated antibodies (AF488 and AF555, from Life technologies, Carlsbad, CA, USA) were used as secondary antibodies. Nuclei were stained with DAPI (Molecular Probes, Eugene, OR, USA). The coverslips were then mounted over slides in AF1 medium (Dako, Agilent, Santa Clara, CA, USA). For live cell imaging experiments, 3 × 10^5^ CHO-GO-D_Ma_-FlAsH or CHO-GO-M_Ma_-FlAsH cells were seeded into each well of a 4-well chamber slide with a 13-mm glass bottom. Twenty-four hours later, the culture media was removed, and each well was washed with HBSS (Hank’s balanced salt solution with Ca^2+^ and Mg^2+^ without phenol red Gibco^®^). Then, the staining solution made of Hoechst 5 µg/mL (nuclear staining, ThermoFisher, Waltham, MA, USA) and FlAsH 1 µM in HBSS was added to each well and incubated for 15–30′ in the cell incubator. After the removal of the staining solution, each well underwent three washing steps in BAL buffer (0.25 mM, ThermoFisher, Waltham, MA, USA). Finally, 250 µL of HBSS was added to each well before confocal imaging. Images were captured using a confocal laser-scanning fluorescence microscope Leica SP5 (Molecular Probes, Leica Microsystem, Wetzlar, Germany) at 63× magnification. Three-dimensional stack images of cells expressing monomeric forms were deconvolved using Huygens Professional software package (version 19.04, Scientific Volume Imaging B.V. Hilversum The Netherlands, http://svi.nl (access on 1 January 2021)). A theoretical point spread function (PSF) was used, choosing the classical maximum likelihood estimation (CMLE) algorithm. Deconvolved images were visualized by twin slicer mode of Huygens, which provides information related to the fluorescence signal and its position inside the cell to show the co-localization of green (AGT) and red (peroxisomes) signals. For qualitative analysis, the ImageJ software was used (http://rsb.info.nih.gov/ij/, 1997–2008, (access on 1 January 2021)). Figure cropping and labelling were performed using Adobe Photoshop.

### 2.13. Fluorescence Recovery after Photo-Bleaching (FRAP)

Fluorescence recovery after photo-bleaching (FRAP) was used to investigate the peroxisomal import kinetics in CHO-GO cells stably expressing D_Ma_-FlAsH and M_Ma_-FlAsH. Cells were plated in 8-well slide ibidi chambers (Ibidi 80826) in complete media. After 48 h, cells were washed in HBSS and incubated in 1 µm FlAsH labelling solution for 20 min at 37 °C with 5% CO_2_. After washing three times in BAL buffer, cells were restored to complete media and directly observed using a Zeiss LSM780 confocal microscope enclosed in a Solent chamber kept at 37 °C with a supply of 5% CO_2_. For the FRAP studies, FlAsH was imaged and bleached using a Plan-Apochromat 63×/1.4 Oil objective with 488 nm laser excitation and emission collected between 500–648 nm. Regions of interest (ROIs) of a fixed dimension enclosing a peroxisome were defined, and pre- and post-bleach images were collected as part of a time series to a maximum of 350 s. Untransfected cells were analyzed as negative control, but their autofluorescent signals were undetectable as compared with the fluorescence signal of transfected samples. One peroxisome was bleached per cell, and at least 20 cells were bleached for each experiment. Data were taken from multiple cells and days for each experiment type. About half the FRAP sets were not analyzed due to organelle movement or loss of focus, but at least 10 were included in computation of each of the recovery time values. The FRAP curves were normalized to 100% for the mean pre-bleached signal. Analysis of the dimer and monomer major alleles (D_Ma_-FlAsH, M_Ma_-FlAsH) was usually performed using SigmaPlot v14 and a simple recovery time constant was obtained by fitting the data with a single exponential rise to the final intensity:

(3) $$A\left(t\right)=\;-a\exp\left[-\frac{\log_{e}\left(2\right)\;t}{T_{\text{½}}}\right]+{\;a}_{0}$$

where A(t) is the bleach signal at times t, a is the pre-exponential value, a_0_ is the eventual recovery level, and T_½_ is the time constant of the bleach recovery. Analysis of some traces of M_Ma_-FlAsH was more complicated due to fading and/or movement of the peroxisomes. Analysis was therefore based on single particle fluorescence analysis, and recovery profiles of either intensity or total signal were fitted by the same exponential rise described by Equation (3), but with the final signal a_0_ replaced by a fading term modelled as an exponential decay:

(4) $$A\left(t\right)=\;-a\exp\left[-\frac{\log_{e}\left(2\right)\;t}{T_{\text{½}}}\right]+a_{0}\;\exp\left[-\frac{\log_{e}\left(2\right)\;t}{T_{0}}\right]$$

where T_0_ is the time constant of the background fading, using iterative non-linear regression. Repeat bleaching experiments were also conducted on D_Ma_-FlAsH and M_Ma_-FlAsH using the same conditions as above but using a larger bleach region encompassing more than one cell. Here, the bleaching was repeated after every second image and with an interval of 20 s between images to allow for recovery.

### 2.14. Statistical Analysis

Experiments were performed at least in triplicate. Statistical analyses were performed using Origin^®^ 8 (Origin Lab) or GraphPad Prism Version 6.0 (GraphPad software, San Diego, CA, USA).

## 3. Results

### 3.1. Biochemical Features of Purified Monomeric and Dimeric AGT

A purified monomeric form of AGT obtained through the interfacial mutations R118A/F238S/F240S on the background of the minor allele (M_Mi_) has been already characterized by our group [32]. Here, we also constructed a monomeric form on the background of the major allele (M_Ma_) by inserting the same interfacial mutations. Size-exclusion chromatography experiments confirmed that the inserted mutations remarkably increased the K_D(dim-mon)_ values of both D_Ma_ and D_Mi_ in both the apo- and the holo-form (Table 1). It should be observed that the effect of the mutations on dimer stability was higher for D_Mi_ than for D_Ma_, thus suggesting that they could somehow synergize with the minor allele polymorphic changes. As a necessary premise for the studies in a cellular system, we compared the biochemical properties of the purified dimeric and monomeric forms of AGT. M_Ma_ and M_Mi_ were folded in solution, as shown by their intrinsic fluorescence emission and far-UV dichroic spectra (Figure S2a and inset) reporting on their tertiary and secondary structure, respectively. As expected, both monomers were catalytically inactive ([44] and Figure S2b), in line with AGT being a functionally-obligate dimer. In addition, they differed from the corresponding dimeric forms for (i) the higher content of hydrophobic surfaces, indicated by their increased fluorescence emission in the presence of the probe ANS (Figure S2c) that binds exposed hydrophobic patches, as previously observed under native and denatured conditions [32,45] and (ii) the lower PLP-binding affinity (Table 1) due to the fact that the coenzyme is held in place by residues coming from both subunits [44]. M_Ma_ and M_Mi_ displayed a sigmoidal thermal-unfolding curve (Figure S2d) but showed a reduced melting temperature as compared with the corresponding dimeric species, in line with spectroscopic data indicating that monomeric AGT is folded but dimerization strongly influences protein stability. Finally, each apoenzyme showed a reduced melting temperature as compared with the corresponding holoenzyme, confirming the structural role of PLP (Table 1).

### 3.2. Expression of Monomeric and Dimeric AGT in Mammalian Cells

**Figure 1:**
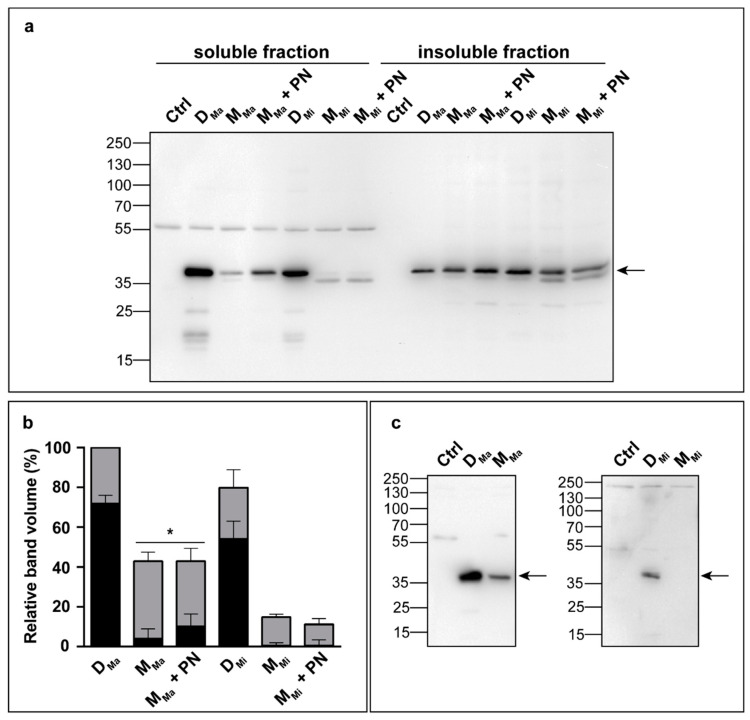
Analysis of AGT protein levels in the absence or presence of pyroxidine (PN). (**a**) Chinese hamster ovary cells stably expressing glycolate oxidase (CHO-GO) untransfected (Ctrl) or expressing the indicated AGT species, treated or not with PN (10 μM) for 7 days, were lysed, and 5 (D_Ma_/M_Ma_) or 10 (D_Mi_/M_Mi_) μg of the soluble and insoluble (as indicated) fractions were analyzed by Western blot with an anti-AGT antibody from rabbit. (**b**) Immunoblot band volumes are represented as histograms and expressed as percentage of the protein present in the soluble (black) and insoluble (grey) fractions of the cell lysate relative to D_Ma_. Data are expressed as mean ± SEM (n = 4). *, *p* < 0.05 (**c**) The soluble fraction of each lysate was analyzed by Western blot using antibodies that specifically recognize the AGT N-terminus (antiPro11 for D_Ma_ and M_Ma_; antiLeu11 for D_Mi_ and M_Mi_). The arrow in panels (**a**,**c**) indicates the band relative to AGT.

To assess how dimerization affects the intracellular fitness of AGT, we expressed D_Ma_, M_Ma_, D_Mi_, and M_Mi_ in a cellular system by using a model of PH1 made up of CHO-GO cells [43], cultured in a low-Vitamin B6 medium to better mimic a physiological extracellular environment [40,47]. As expected, cells expressing D_Ma_ and D_Mi_ displayed AGT specific activity values of 150 ± 5 and 114 ± 8 nmol/min/mg protein, respectively, while no transaminase activity could be detected in lysates of cells expressing M_Ma_ and M_Mi_, in line with the results on purified proteins. Cross-linking experiments (Figure S3a) confirmed that AGT was present as a dimer in cells expressing D_Ma_ and D_Mi_. Under the same conditions, M_Ma_ was mostly present in a 37 kDa monomeric form, with faint bands at the level of the dimer and at higher molecular weight, while only a faint band at an apparent molecular weight < 37 KDa was detected in cells expressing M_Mi_. To further investigate the cellular fate of monomeric AGT, protein levels were evaluated in the soluble and insoluble fractions of cells expressing D_Ma_, M_Ma_, D_Mi_, or M_Mi_ (Figure 1a,b). The total amount of M_Ma_ and M_Mi_ was reduced to 43% and 19% of D_Ma_ and D_Mi_, respectively, and more than 75% of the protein was present in the insoluble fraction. In addition, a band at approximately 35 kDa was present in the lysate of cells expressing D_Mi_ and M_Mi_, which represents a truncated form obtained upon N-terminal cleavage, as confirmed by using antibodies specifically targeting the N-terminus (Figure 1c). Addition of pyridoxine (PN), a precursor of PLP used in clinics and as a food additive [48], increased the levels of soluble M_Ma_ (Figure 1a,b) and led to the appearance of dimeric species in cross-linking experiments (Figure S3b). In order to increase the population of dimeric species in cells expressing M_Ma_ in the presence of PN, we treated with a 50× molar excess of cross-linker due to the very low efficiency of the cross-linking process already observed in the same cellular system [49]. Under the latter conditions, the band of the monomer in the absence of PN was no more visible because the equilibrium was shifted toward aggregates. Nevertheless, these data are in line with the results obtained with the purified protein, indicating that PLP binding promotes dimerization [32] and that holoAGT is less prone to aggregation [50]. No effects were observed when the same treatment was performed in cells expressing M_Mi_. Since M_Mi_ displays a K_D(dim-mon)_ value higher than M_Ma_ (Table 1), it can be hypothesized that it is unable to dimerize even in the presence of the high intracellular PLP concentrations resulting from PN treatment. Therefore, the artificial monomeric forms of AGT tend to undergo aggregation and proteolytic degradation. The aggregation of M_Ma_ can be counteracted by the administration of exogenous coenzyme that induces dimerization. In the case of M_Mi_, since the full-length form is present at very low levels and no effect is observed in the presence of PN, we considered only D_Ma_ and M_Ma_ for the subsequent experiments.

**Figure 2:**
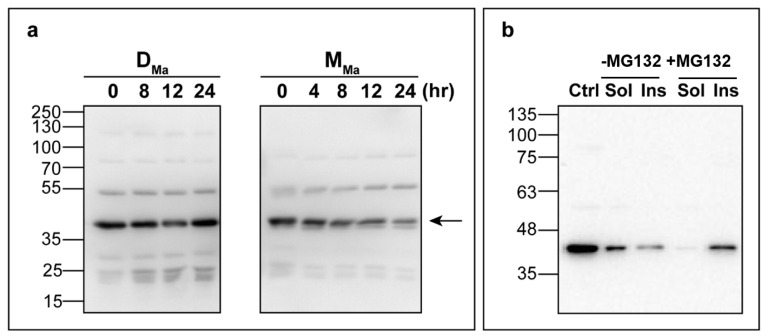
Intracellular stability of monomeric and dimeric AGT. (**a**) Measurement of the half-life of D_Ma_ and M_Ma_ by a cycloheximide chase assay. CHO-GO cells expressing either D_Ma_ or M_Ma_ were grown in the presence of cycloheximide (10 µg/mL); at different times cells were harvested and lysed. Five micrograms of cell lysates were analyzed by Western blot with an anti-AGT antibody from rabbit. The arrow indicates the band relative to AGT (**b**) Effect of the treatment with MG132 on the AGT expression levels of CHO-GO expressing M_Ma_. CHO-GO cells expressing M_Ma_ were treated for 24 h with 10 µM MG132 and 10 μg of lysate were analyzed by Western blot for the amount of AGT in the soluble (Sol) and insoluble (Ins) fraction using an antibody against AGT from rabbit. Ctrl represents the positive control of CHO-GO cells expressing D_Ma._ Images shown are representative of at least three independent experiments.

We first compared the intracellular stability of M_Ma_ with that of D_Ma_ by a cycloheximide chase assay (Figure 2a). While D_Ma_ levels were stable up to 24 h, we observed a time-dependent decrease of the M_Ma_ levels. By fitting the densitometric data as a function of time, we calculated the half-life of the protein, which was found to be 9.1 ± 0.9 h. To investigate the mechanisms underlying the reduced half-life of M_Ma_, we treated cells with (i) MG132, a well-recognized inhibitor of the ubiquitin-proteasome system (UPS), or (ii) chloroquine, a molecule that prevents lysosomal degradation and could report on possible microautophagy or pexophagy events [48,49]. While treatment with chloroquine caused no effect, MG132 reduced the soluble levels of monomeric AGT while promoting an accumulation of the protein in the insoluble fraction (Figure S4 and Figure 2b). This result is consistent with the hypothesis that UPS could be involved in the degradation of partly-folded monomeric intermediates that accumulate in the cytosol and self-aggregate upon proteosomal inhibition.

Altogether, our results indicate that, when expressed in eukaryotic cells, M_Ma_ and M_Mi_ do not display any transaminase activity and are expressed at reduced levels as compared to D_Ma_ and D_Mi_. Two factors contribute to the latter effect: (i) the increased tendency to aggregation and (ii) the higher susceptibility to undergo proteolytic cleavage at the N-terminus, which reduces the intracellular half-life of the protein by possibly stimulating degradation by the UPS. All the effects attributed to monomerization are potentiated by the presence of the minor allele polymorphic mutations but reduced by the addition of exogenous coenzyme.

### 3.3. Subcellular Localization of Monomeric and Dimeric AGT

**Figure 3:**
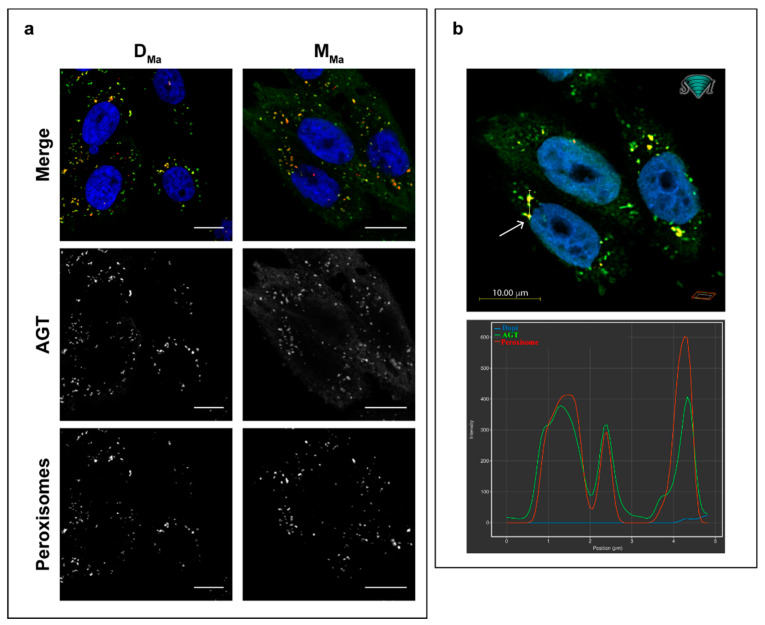
Subcellular distribution of monomeric and dimeric AGT. (**a**) CHO-GO cells expressing either D_Ma_ or M_Ma_ were fixed and stained with antibodies against AGT (green) and peroxisomal protein catalase (red). Nuclei were stained with Dapi (blue). Merged and single channel images come from a single z-plane. Scale bar: 10 µm. (**b**) Deconvolution of IFM images for M_Ma_ shown in panel (a) along with the corresponding RGB profiles.

D_Ma_ showed a clear peroxisomal localization in CHO-GO cells, as revealed by immunofluorescence microscopy (Figure 3a). Intriguingly, we also detected the presence of peroxisomal AGT in cells expressing M_Ma_, indicating that monomers are able to be imported in the peroxisomal matrix, as confirmed by the image deconvolution analysis and RGB profiles shown in Figure 3b, where the AGT signal colocalizes with that of catalase. However, part of the protein showed a cytosolic localization, indicating that the oligomeric state can influence the subcellular localization of AGT by reducing the efficiency of the peroxisomal import. However, since immunofluorescence microscopy (IFM) experiments are performed in fixed cells, they give a snapshot of the steady-state intracellular localization of the protein without any information on the import kinetics.

We, therefore, resorted to live-cell confocal microscopy and used the technology based on the fluorescent dye FlAsH-EDT_2_ to label AGT without any impact on the structure and/or the intracellular behavior of the protein. FlAsH-EDT_2_ is a membrane-permeable dye that emits fluorescence upon specific binding to the CCXXCC motif [51]. We engineered AGT by inserting the CCPGCC sequence between Asp244 and Asp245, an exposed region whose alteration is not predicted to affect the enzyme functional and structural properties, as previously reported by our group [52]. When compared to the corresponding unmodified form, recombinant purified D_Ma_-FlAsH and M_Ma_-FlAsH displayed similar kinetic and coenzyme binding properties (Table S1), thus indicating that the inserted CCPGCC sequence did not alter the overall structural and functional properties of the enzyme. As expected, the insertion of the R118A/F238S/F240S interfacial mutations on D_Ma_-FlAsH completely abolished catalytic activity and reduced coenzyme binding affinity [32]. In addition, D_Ma_-FlAsH displayed thermal stability parameters analogous to those shown in Table 1 and to those previously reported for D_Ma_ [39], with a similar denaturation enthalpy and only a 4 °C reduction of the T_m_ value (Table S2). Since it is known that the use of reducing agents does not affect AGT thermal stability [39], these data indicate that the FlAsH-EDT_2_ recognition sequence has little effect on AGT stability and denaturation mechanism. As for M_Ma_-FlAsH, the finding that it displays a strongly reduced thermal stability is in line with previous data on monomeric AGT [32] and those shown in Table 1, indicating a T_m_ value reduced by 22–24 °C with respect to the corresponding dimeric form. We also tested the interaction of the two forms with the Pex5p carrier and found that the insertion of the FlAsH recognition sequence did not prevent binding (Table S3, Figure S5), and that the binding parameters of the monomeric and dimeric form are similar, thus, supporting the idea that dimerization is not critical for binding. Finally, we verified that the FlAsH-EDT_2_ dye emits fluorescence at 533 nm (excitation at 508 nm) upon binding to D_Ma_-FlAsH, and that the signal is stable for at least 5 min. This is in line with the high affinity and specificity, as well as with the stability of binding previously observed [53].

When expressed in CHO-GO cells, M_Ma_ and M_Ma_-FlAsH displayed an analogous quaternary assembly, although the insertion of the CCPGCC sequence caused a reduction of the AGT levels (Figure S6). It can be hypothesized that the inserted sequence increases the flexibility of the surface loop 251–266 and makes the protein more susceptible to degradation inside the cell. In agreement with the data obtained in fixed cells expressing the untagged forms, in live cell imaging experiments on cells expressing D_Ma_-FlAsH, the FlAsH-EDT2 dye generated a pattern consistent with the peroxisomal localization of dimeric AGT, while in cells expressing M_Ma_-FlAsH, the strong fluorescence associated with peroxisomes was accompanied by a diffuse fluorescent signal in the cell cytosol, confirming the partial cytosolic localization of the artificial monomeric forms (Figure S6).

Overall, these data validate the use of FlAsH-tagged forms as models for live cell imaging experiments.

### 3.4. Peroxisomal Import Kinetics of Monomeric and Dimeric AGT

**Figure 4:**
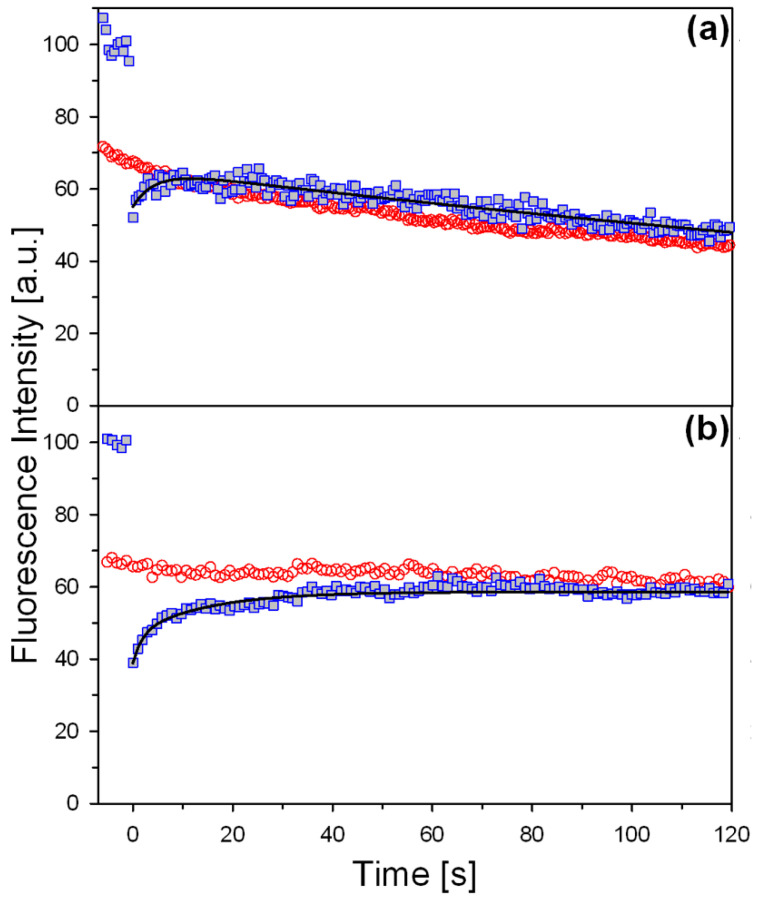
Fluorescence recovery after photo-bleaching (FRAP) curves. Graphs showing the recovery of fluorescence as a function of time of (**a**) M_Ma_, (**b**) D_Ma_. Each panel shows a Scheme 4 (panel (**a**)) or 3 (panel (**b**)), and a control area from a non-bleached region of interest showing acquisitional bleach (red circles). The intensity axis has been normalized to the pre-bleach average.

We measured the peroxisomal import rate of D_Ma_-FlAsH and M_Ma_-FlAsH by studying the recovery of the fluorescence after photo-bleaching a whole peroxisome. Considering that no free dye was present during the experiment, that the dye emitted fluorescence only when it was bound to the CCPGCC motif, and that the signal was stable during the timescale of the FRAP experiment, we can be confident that the recovery of fluorescence is associated with the presence of new AGT-FlAsH molecules, thus, informing on the kinetics of import (Movies S1 and S2). As a control, we first analyzed whether the fluorescence outside of the bleached area dropped after repeated bleaching, to demonstrate that the system is able to return to equilibrium. As shown in Figure S7 and Movies S3 and S4, we repeatedly bleached a large area over more than one cell and could show that the regions of interest (ROIs) in the bleached cells but outside of the bleach region also lost fluorescence, not through dark state nor through acquisitional bleaching. Thus, equilibrium was restored as the bleached ROI proteins were able to exit and equilibrate within the cell, a finding that further corroborates that the fluorescence recovery after bleaching is due to an import of particles into peroxisomes and not to aggregates. In preliminary experiments, we also found that, during image acquisition, there was a progressive slight loss of fluorescent signal. Thus, in all FRAP experiments, we registered the fluorescence of two regions: the bleached ROI corresponding to a peroxisome, to determine the kinetics of import, and a non-bleached control ROI (Figure 4). The latter is fundamental to understand whether the recovery of fluorescence in the bleached region has plateaued. The signal corresponding to the peroxisome showed a rapid recovery after bleaching, which stabilized in less than 30 s once equilibrium had been reached. From these data, we could estimate that D_Ma_-FlAsH and M_Ma_-FlAsH display identical time constants (2.5 ± 0.3 s vs. 2.5 ± 0.4 s, respectively). This would indicate that the oligomeric state of the protein does not influence import kinetics.

## 4. Discussion

In this work, we performed a detailed analysis of the role played by the quaternary structure for the fitness and subcellular localization of a peroxisomal matrix protein in a live cellular system. We used, as model human AGT, a dimeric enzyme whose deficit is associated with the rare disease PH1 [25]. AGT folds in the cell cytosol and is then targeted to peroxisomes by a C-terminal PTS1 KKL sequence [54]. AGT contains one of the weakest PTS1 sequences known for human peroxisomal proteins, as already reported and confirmed by our data (Table S3), but it is efficiently imported due to the high protein levels [55,56]. Consequently, any event that may sequester AGT from the cytosolic pool available for interaction with Pex5 could prevent its proper import. The current model of the AGT folding pathway postulates the formation of monomeric intermediates that first generate an apodimer, which subsequently binds PLP and is imported to peroxisomes. Along this pathway, partially folded states can interact with molecular chaperones [29,31,57]. Dimerization likely plays a crucial role in the folding and correct peroxisomal import of the protein [30], thus, making AGT an ideal model to investigate the interplay between folding, dimerization, and import.

Herein, we have tested this interplay by comparing the behavior of the native dimeric and artificial monomeric forms of AGT in mammalian cells, as well as the influence of a polymorphism that makes the protein susceptible to mitochondrial mistargeting [27]. Our data demonstrate that interfering with dimerization strongly impacts the intracellular behavior of AGT in many respects. First, artificial monomeric AGT is more susceptible to degradation than the corresponding dimer, in line with its reduced conformational stability. We can hypothesize that proteolytic degradation is triggered by both the exposure of hydrophobic surfaces at the monomer–monomer interface and the presence of a highly flexible N-terminal tail, which wraps the neighboring subunit in the dimer but becomes exposed to cytosolic proteases in the monomer [39,49,58]. Indeed, Western blot data indicate that monomeric AGT is prone to proteolytic cleavage at the N-terminus. Second, artificial monomeric AGT tends to aggregate, as indicated by Western blot experiments. In addition, immunofluorescence microscopy experiments, in both fixed and live cells expressing M_Ma_, suggest partial protein accumulation in the cytosol. It is known that purified AGT has a strong tendency to aggregate by processes driven by partial unfolding, hydrophobic interactions, and/or electrostatic forces [31,50,57]. Although it is difficult to identify the main driving force for intracellular aggregation, exposed hydrophobic surfaces of the interface are likely to play a major role.

Notwithstanding the major impact of dimerization on protein folding and stability, we unexpectedly found that monomeric forms can still be imported into peroxisomes, although with lower efficiency as a partial cytosolic localization was clearly present. The latter phenomenon, observed in both fixed and live cells, does not depend on an impaired interaction with the Pex5p carrier, nor different kinetics of import, as revealed by the use of the FRAP technique. To the best of our knowledge, this paper reports the first application of the FRAP technique to measure the import kinetics of a matrix peroxisomal protein in live cells, with the major advantage over previous work to avoid incorporation of fluorescent tags that may affect intracellular behavior and interaction with the import machinery. Rather, AGT was engineered to interact with the FlAsH dye that is suitable for live cell analyses [59]. Indeed, the FlAsH target sequence was added to an AGT external loop previously used for imaging studies [52], and we verified that the modification does not interfere with the molecular properties of each enzymatic form under study, although it reduces intracellular protein levels. These experiments revealed that the import of AGT is very rapid and occurs with kinetics in a time scale of seconds. This rate appears to be higher than that previously reported for other peroxisomal proteins, such as catalase, dihydroxyacetone synthase, or urate oxidase [16,60,61,62], and the use of indirect assays or in vitro transcription/translation systems, rather than a direct fluorescence measure, may account for some of these discrepancies.

Notably, FRAP experiments suggest that monomeric and dimeric AGT have similar import kinetics. It must be pointed out that our approach does not allow a direct visual discrimination between the two forms. Nevertheless, cross-linking experiments on the total cell lysate (Figure S3a) clearly indicate that M_Ma_, with or without the FlAsH target sequence, should be present as monomers. Thus, we can suggest that monomeric and dimeric AGT could be actually imported as monomers and dimers, respectively. Following this view, the peroxisomal import machinery does not seem to show a preference based on the AGT quaternary structure, in line with previous reports [63]. From a structural-thermodynamic point of view, this agrees with the finding that the principal and ancillary targeting sequences of AGT are not part of the monomer–monomer interface [54,56], thus, indicating that there is no obvious competition between Pex5p binding and AGT oligomerization. Previous studies have focused on the equilibrium of the process, i.e., on the ability of Pex5p to recognize monomeric or oligomerized cargoes, as well as on the role of Pex5p binding in counteracting oligomerization, and contrasting results have been reported. On the one hand, some studies have shown that peroxisomes can import oligomerized proteins, mainly based on the evidence that some matrix proteins are imported as hetero-oligomers by a piggy-back pathway [18,19]. On the other hand, however, some proteins are imported as monomers with Pex5p binding blocking oligomerization [12,20,21], and the idea that the peroxisomal import machinery shows a preference for monomeric substrates is largely supported in the case of homo-oligomers. Our results support a model in which the checkpoint for the import of AGT is not the interaction with the carrier, which occurs with similar kinetics and equilibrium for monomeric and dimeric forms, but rather the efficiency of cargo folding in the cytosol. Indeed, the population of monomeric folding intermediates may undergo distinct events, which include possible degradation, aggregation, dimerization, and peroxisomal import [57,63]. Under normal physiological conditions, it is likely that the rate of AGT dimerization far exceeds that of aggregation or degradation so that the protein is probably imported as a holodimer into peroxisomes. If interference with dimerization occurs, the population of monomeric intermediates is increased and becomes available for events other than import, with the final result to reduce the amount of functional intraperoxisomal AGT. On the contrary, the treatment with molecules that promote dimerization shifts the balance away from degradation and aggregation in favor of oligomerization and correct folding. One example is the treatment with PN, which leads to the intracellular accumulation of PLP. Many studies of our group and others have highlighted how the coenzyme is able to promote AGT dimerization [39,40,64,65], an effect explained by the fact that the enzyme active site is made up of residues coming from both subunits, and thus, coenzyme binding also stabilizes monomer–monomer interactions [32]. Nevertheless, our study suggests that coenzyme administration, in the form of PN, is able to act on monomeric AGT and induce dimerization. This model also reconciles the findings that monomeric AGT forms generate aggregates probably stabilized by hydrophobic interactions, while truncated AGT that undergoes a normal dimerization generates intraperoxisomal aggregates stabilized by electrostatic forces [49]. This view is reinforced by the finding that the import would locally concentrate the protein, a condition that strongly favors aggregation from dimeric native or native-like species but would prevent aggregation from monomeric species by stabilizing the dimer [50]. In this scenario, the minor allele polymorphism makes monomeric AGT more susceptible to degradation and aggregation. This is probably due to the P11L mutation that increases the flexibility of the N-terminal arm, which becomes more prone to intracellular cleavage and destabilizes the dimer [41,45,57].

Overall, our data reveal that folding kinetics of cargo proteins in the cytosol may play a key role in peroxisomal import of matrix proteins, thus, contributing to the overall efficiency of the import process. In this regard, it cannot be excluded that peroxisomal import/cytosolic accumulation could also represent a mechanism to regulate the activity of enzymes, as previously reported for inducible nitric oxide synthase [66]. It is also worth speculating that evolutionary divergence in consensus amino acids along mammalian speciation could lead to changes in folding/unfolding kinetics in the cytosol, thus, contributing to the differences in intracellular location observed among AGT proteins from mammalian species [67].

Our results also provide insights into the pathogenesis of PH1, which is considered a misfolding disease because most pathogenic missense mutations reduce the efficiency of the AGT folding pathway [68]. We can suggest that mutations increasing the population of monomeric intermediates likely result in enhanced cytosolic degradation, aggregation, and eventual mistargeting if associated with the minor allele. Particularly, the structural and folding perturbations caused by the most common PH1 mutations (such as G170R, F152I, and I244T) may cause simultaneously aggregation and mistargeting of the enzyme [41,69,70], and the preference of a given mutation for one or the other mechanism may depend on how efficiently the energetic perturbation on the dimeric and monomeric forms of AGT relevantly translate into the rate-limiting states for each pathogenic mechanism. Interestingly, these mutations are also clinically responsive to PN administration, in line with our data, which definitely prove that PN is able to rescue for dimerization and stability impairments. On the other hand, the effects of mutations resulting exclusively in the formation of intraperoxisomal aggregates, such as the Gly41 variants [58], probably cause less pronounced effects on dimerization kinetics, but are more related to conformational changes of the dimeric form, making the protein prone to self-association upon efficient import into the peroxisomal matrix.

## Figures and Tables

**Table 1:** Parameters for quaternary structure, pyridoxal 5′-phosphate (PLP) binding and thermal denaturation of alanine:glyoxylate aminotransferase (AGT) enzymes. Experiments were performed in KP 0.1 M, pH 7.4. Values of K_D(PLP)_ and T_m_ of M_Ma_ and M_Mi_ were determined at a protein concentration in which the monomeric form was >90%. Tm values were obtained by monitoring the loss of circular dichroism (CD) signal at 222 nm. Data on the holo-forms were determined in the presence of saturating PLP.

Enzymatic species	K_D(dim-mon)_ Apo-Form (μM)	K_D(dim-mon)_ Holo-Form (μM)	K_D(PLP)_ (μM)	T_m(apo)_ (°C) ^a^	T_m(holo)_ (°C)
D_Ma_	<0.3	<0.3	0.27 ± 0.03	61.1 ± 0.3 66.4 ± 0.3	77.5 ± 0.3
M_Ma_	4.1 ± 0.3	1.5 ± 0.3	3.2 ± 0.6	54.6 ± 0.3	54.9 ± 0.3
D_Mi_	<0.3	<0.3	0.26 ± 0.02	53.8 ± 0.3 66.1 ± 0.3	73.6 ± 0.3
M_Mi_	80 ± 1	9.4 ± 0.5	4.4 ± 0.5	47.3 ± 0.3	50.1 ± 0.1

^a^ The two T_m_ values of the D_Ma_ and D_Mi_ species have been attributed to the unfolding of the large and small domains of AGT (see ref. [46]).

## Data Availability

The data presented in this study are available on request from the corresponding author.

## Supplementary Materials

The following are available online at https://www.mdpi.com/article/10.3390/jpm11040273/s1, Figure S1: Sequence alignment of the AGT species under study, Figure S2: Spectral, kinetic, and stability properties of dimeric and monomeric AGT. Figure S3: Cross-linking analyses of the total extract of CHO-GO cells expressing D_Ma_, M_Ma_, D_Mi_, or M_Mi_, Figure S4: Effect of the treatment with MG132 or chloroquine on the expression levels of M_Ma_, Figure S5: ITC titrations of D_Ma_ and M_Ma_ with Pex5-pdb, Figure S6: Expression, oligomeric state, and subcellular localization of D_Ma_-FlAsH and M_Ma_-FlAsH in CHO-GO cells, Figure S7: Control FRAP curves, Table S1: Comparison of the kinetic parameters and PLP binding affinity of AGT forms under study in the absence or presence of the FlAsH binding sequence title, Table S2: Stability parameters for AGT variants as derived from DSC data and fitting to a two-state kinetic model, Table S3: Thermodynamic binding parameters for the interaction of Pex5p-pbd with AGT enzymes, Movie S1: FRAP (fluorescence recovery after photobleaching) of D_Ma_-FlAsH, Movie S2: FRAP of M_Ma_-FlAsH, Movies S3 and S4: Control FRAP (fluorescence recovery after photo-bleaching) experiments.
