# Supplementary material for: Dimerization Drives Proper Folding of Human Alanine:Glyoxylate Aminotransferase But Is Dispensable for Peroxisomal Targeting
Source: J Pers Med. 2021 Apr 6;11(4):273. doi: 10.3390/jpm11040273 (PMC8067440; doi:10.3390/jpm11040273)
Supplement: Supplementary file 1 [file jpm-11-00273-s001.zip › jpm-1155181 supplementary materials/jpm-1155181 supplementary.docx]

**SUPPLEMENTARY INFORMATION**

**Dimerization drives proper folding of human alanine:glyoxylate aminotransferase but is dispensable for peroxisomal targeting**

Mirco Dindo^1,A,B^, Giulia Ambrosini^2,A^, Elisa Oppici^2^, Angel L. Pey^3^, Peter J. O’Toole^4^, Joanne L. Marrison^4^, Ian E.G. Morrison^4^, Elena Butturini^2^, Silvia Grottelli^1^, Claudio Costantini^1^ and Barbara Cellini^1^*

^1^ *Department of Experimental Medicine, University of Perugia, Perugia, Italy*

^2^ *Department of Neurosciences, Biomedicine and Movement Sciences, University of Verona, Verona, Italy*

*^3^ Departamento de Química Física, Unidad de Excelencia de Química aplicada a Biomedicina y Medioambiente e Instituto de Biotecnología, Facultad de Ciencias, Universidad de Granada, 18071 Granada, Spain; ^4^ Bioscience Technology Facility, Department of Biology, University of York, York, YO23 3GE, UK*

^A^ The authors contributed equally to this work

^B^Present address: Okinawa Institute of Science and Technology Graduate University, 1919-1 Tancha, Onna-Son, Okinawa 904-0412 Japan

*Corresponding author: Prof. Barbara Cellini, E-mail: [barbara.cellini@unipg.it](mailto:barbara.cellini@unipg.it)


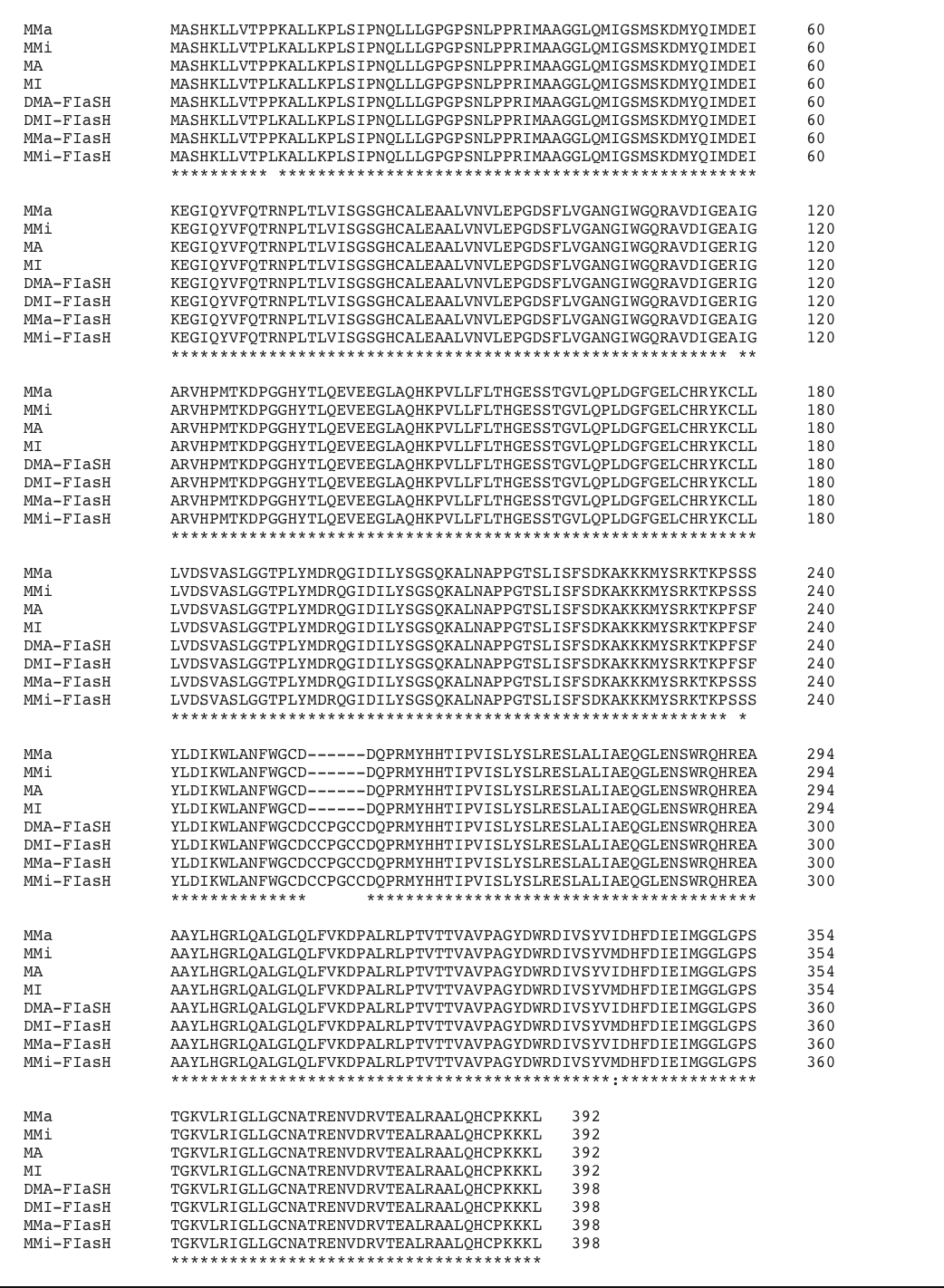


**Figure S1. Sequence alignment of the AGT species under study.** Polymorphisms of the minor allele, mutated interfacial residues, and the FlAsH target sequence are highlighted with red, blue, and green boxes, respectively. The figure was rendered using ClustalW.

**Figure S2. Spectral, kinetic, and stability properties of dimeric and monomeric AGT.** (a) Intrinsic fluorescence emission spectra (exc. 280 nm) at 1 μM concentration; inset: far-UV CD spectra. (b) Enzymatic activity assay of D_Ma_ and M_Ma_. The image shows the decrease of absorbance at 340 nm due to NADH consumption during the conversion of pyruvate produced by the D_Ma_ (⎯) and M_Ma_ (- - -) to lactate by LDH. The red arrow indicates the start of the assay upon LDH addition. The total change of absorbance at 340 nm is proportional to AGT activity. (c) Emission fluorescence spectra (exc. 365 nm) of 8-anilino-1-naphthalenesulfonic acid (ANS) in the presence of the analyzed species at 1 μM concentration. The increase in fluorescence of the ANS probe is indicative of an increased exposure of hydrophobic surfaces. All measurements were obtained in 0.1 M KP, pH 7.4. (⎯), D_Ma_; (- - -), M_Ma_; (- ⋅ - ⋅ -), D_Mi_; (⋅ ⋅ ⋅), M_Mi_. (d) Thermal unfolding curves of D_Ma_ and M_Ma_ obtained by monitoring the loss of the CD signal at 222 nm. The black lines indicate the fitting of the data to a sigmoidal two-state denaturation curve.


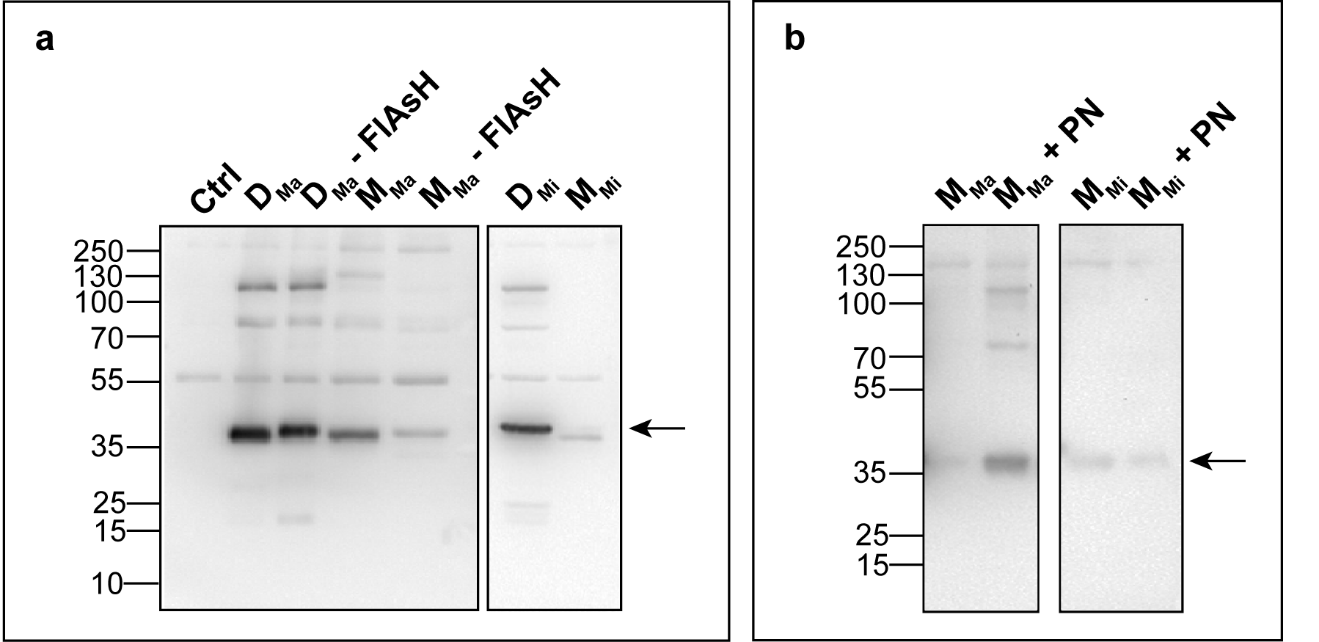


**Figure S3. Cross-linking analyses of the total extract of CHO-GO cells expressing D_Ma_, M_Ma_, D_Mi_ or M_Mi_.** CHO-GO cells untransfected (Ctrl) or expressing the indicated AGT forms were cultured in low-B6 medium (a) or in the presence or absence of 10 μM pyridoxine (PN) (b), lysed and cross-linked with BS(PEG)_5_ at 5X (a) or 50X (b) molar excess; 5 (D_Ma_ and M_Ma_) or 10 (D_Mi_ and M_Mi_) μg of lysate were analyzed by Western blot with an anti-AGT antibody from rabbit. The band of the monomer is indicated by an arrow. As already reported ([1,2], whole extracts of CHO cells expressing AGT upon cross-linking with BS(PEG)_5_ display two bands of dimeric species at approximately 75 and 110 kDa, which probably represent two conformations of the dimer showing a different electrophoretic mobility. Images shown are representative of three independent experiments. The arrows indicate the band relative to AGT.

**
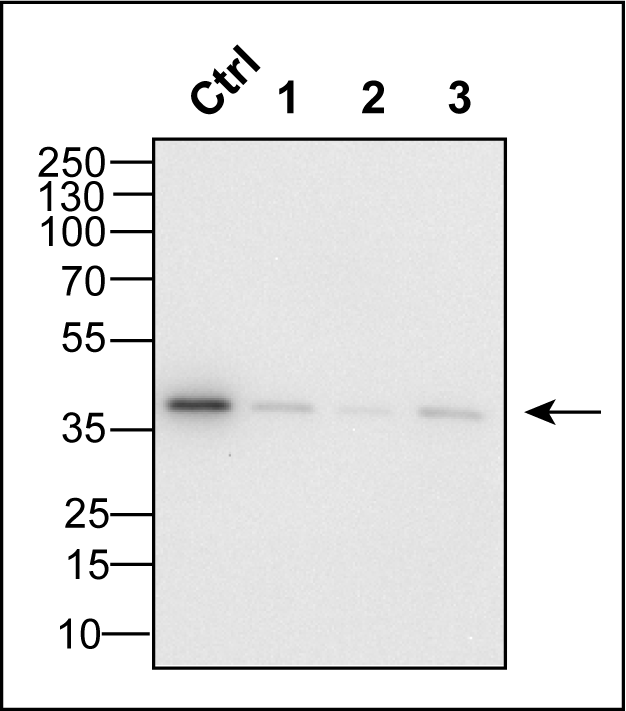
**

**Figure S4. Effect of the treatment with MG132 or chloroquine on the expression levels of M_Ma_.** CHO-GO cells expressing M_Ma_ were treated with MG132 or chloroquine for 24h and then lysed. 5 μg of soluble cell lysate were loaded on SDS/PAGE and transferred on nitrocellulose membrane. The membrane was then immunoblotted with: anti-AGT from rabbit (1:6000), The immunoblot lanes are coded as follow: 1 untreated cells; 2 cells treated with MG132 (10 µM); 3 cells treated with chloroquine (50 µM). Ctrl represents the positive control of CHO-GO cells expressing D_Ma_. The image shown is representative of at least 3 independent experiments. The arrow indicates the band relative to AGT.





D_MaFlAsH_

M_MaFlAsH_

**Figure S5. ITC titrations of D_Ma_ and M_Ma_ with Pex5-pdb.** Experiments were carried out at 10°C in Na-Hepes 20 mM NaCl 200 mM pH 7.4 and 0.2 mM β-mercaptoethanol. Solutions containing D_Ma_ or M_Ma_ (9-11 μM) were titrated with Pex5p-pbd (290 μM). Blank titrations were carried out likewise but without AGT proteins in the cell. Raw data were integrated, and upon subtraction of corresponding heats from blank titrations, isotherms were analyzed according to a single-type of independent binding sites model. Number of sites was fixed to 1 due to low affinities.

**
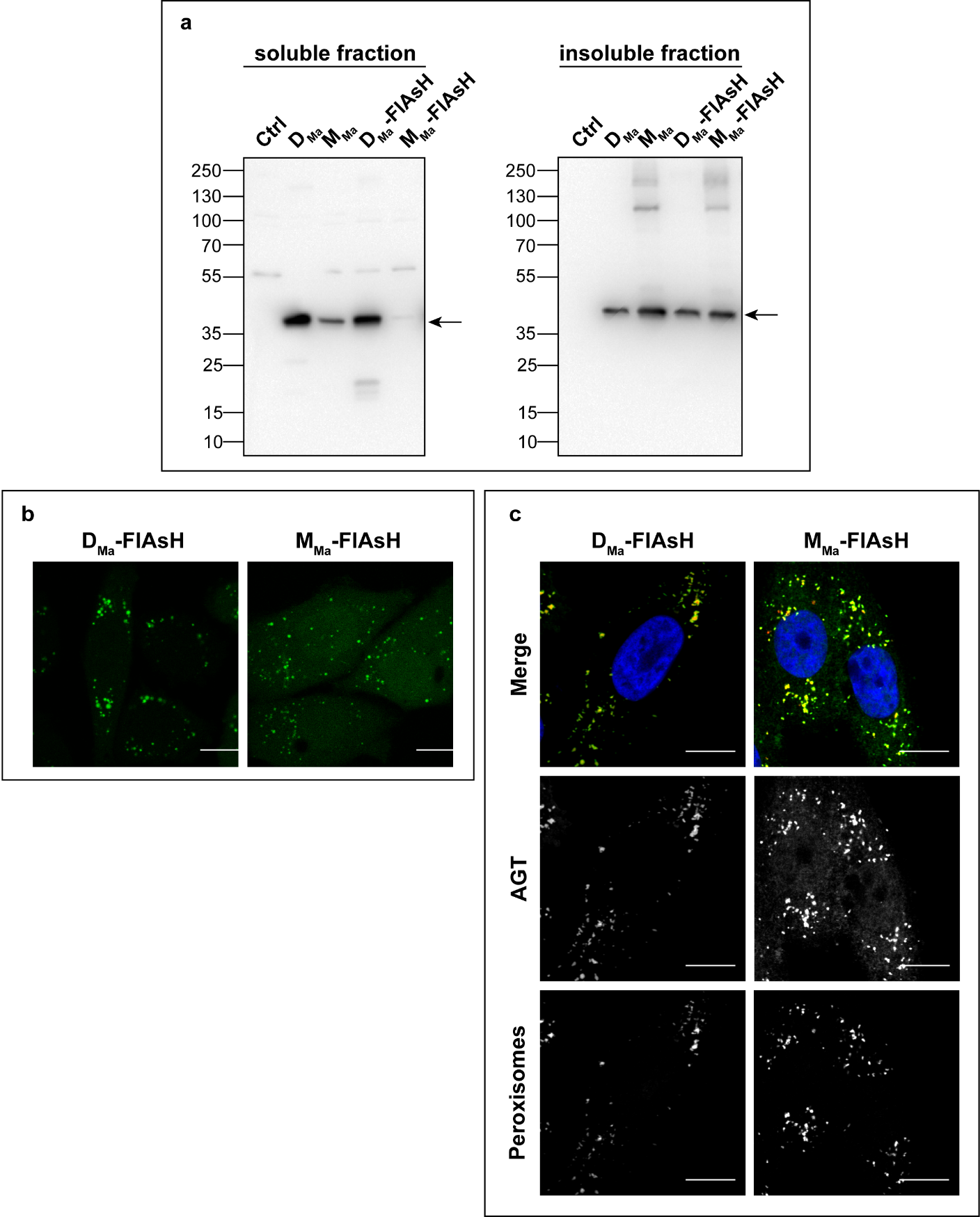
**

**Figure S6. Expression, oligomeric state and subcellular localization of D_Ma_-FlAsH and M_Ma_-FlAsH in CHO-GO cells.** (a) CHO-GO cells untransfected (Ctrl) or expressing the indicated AGT forms were lysed and the soluble (left panel) and insoluble (right panel) fractions of the cell lysate were analysed by Western blot with an anti-AGT antibody from rabbit. The arrow indicates the band relative to AGT. (b) 24 h after seeding, CHO-GO cells expressing D_Ma_-FlAsH or M_Ma_-FlAsH were stained with the FlAsH labelling solution (green). (d) CHO-GO cells expressing D_Ma_-FlAsH or M_Ma_-FlAsH were fixed and stained with antibodies against AGT (green) and peroxisomal proteins (red). Nuclei were stained with Dapi (blue). Merge and single channel images come from a single z-plane. Scale bar: 10 µm. Images shown are representative of three independent experiments.

**a**

**b**

**Figure S7. Control FRAP curves.** Graphs showing the repeated bleaching and recovery of fluorescence as a function of time of selected regions of interest (ROIs) in cells expressing D_Ma_ (a), and M_Ma_ (b), D_Mi_. ROI1: bleached area over more than one cell; ROI2, 3, and 4: areas in the bleached cells outside of the bleached region; ROI5: non-bleached control cell.

**Legend for Movie S1. FRAP (fluorescence recovery after photobleaching) of D_Ma_-FlAsH.**

48 h after seeding cells were incubated for 20 minutes with the FlAsH labelling solution, washed three times in BAL buffer and used in the FRAP experiments as indicated in the Material and Methods section. The movie shows a time series covering the pre-bleach, bleach and post-bleach time points of a typical D_Ma_-FlAsH FRAP. Data for analysis were taken from a bleached peroxisome (white square) and a control non-bleached peroxisomal region of interest (white circle). Images were taken every second for 100 seconds.

Scale bar: 5μm.

**Legend for Movie S2. FRAP of M_Ma_-FlAsH.**

48 h after seeding cells were incubated for 20 minutes with the FlAsH labelling solution, washed three times in BAL buffer and used in the FRAP experiments as indicated in the Material and Methods section. The movie shows a time series covering the pre-bleach, bleach and post-bleach time points of a typical M_Ma_-FlAsH FRAP. Data for analysis were taken from a bleached peroxisome (white square) and a control non-bleached peroxisomal region of interest (white circle). Images were taken every second for 100 seconds.

Scale bar: 5μm.

**Legend for Movie S3. Control FRAP (fluorescence recovery after photobleaching) experiments.** Recovery of fluorescence as a function of time of selected regions of interest (ROIs) in cells expressing D_Ma_. ROI1: bleached area over more than one cell; ROI2, 3, and 4: areas in the bleached cells outside of the bleached region; ROI5: non-bleached control cell.. The time series was continued for 1920 sec (32min): the first 5 images were taken every 20 seconds, followed by a repeated bleach after every 2 images with 20 sec intervals between images. Scale bar: 10μm.

**Legend for Movie S4. Control FRAP (fluorescence recovery after photobleaching) experiments.** Recovery of fluorescence as a function of time of selected regions of interest (ROIs) in cells expressing M_Ma_. ROI1: bleached area over more than one cell; ROI2, 3, and 4: areas in the bleached cells outside of the bleached region; ROI5: non-bleached control cell. The time series was continued for 1920 sec (32min): the first 5 images were taken every 20 seconds, followed by a repeated bleach after every 2 images with 20 sec intervals between images. Scale bar: 10μm.

**Table S1. Comparison of the kinetic parameters and PLP binding affinity of AGT forms under study in the absence or presence of the FlAsH binding sequence**. Experiments were performed in 0.1 M KP, pH 7.4 at 25°C.

| **Enzymatic species** |  | **Substrate** | **Cosubstrate** | **k_cat_**  **(s^-1^ )** | **K_M_**  **(mM)** | **k_cat_/K_M_**  **(mM^-1^s^-1^)** | **K_D(PLP)_**  **(μM)** |
| --- | --- | --- | --- | --- | --- | --- | --- |
| **D_Ma_** |  | L-alanine  Glyoxylate | Glyoxylate  L-alanine | 45 ± 2  45 ± 3 | 31 ± 4  0.23 ± 0.05 | 1.4 ± 0.2  196 ± 44 | 0.27 ± 0.03 |
| **D_Ma_-FlAsH** |  | L-alanine  Glyoxylate | Glyoxylate  L-alanine | 40 ± 1  40 ± 1 | 45 ± 3  0.40 ± 0.04 | 0.88 ± 0.07  100 ± 10 | 0.25 ± 0.03 |
| **M_Ma_-FlAsH** |  | L-alanine  Glyoxylate | Glyoxylate  L-alanine | n.d. | n. d. | n. d. | 7.2 ± 1.1 |

**Table S2. Stability parameters for AGT variants as derived from DSC data and fitting to a two-state kinetic model**. Experiments were performed in Na-HEPES 20 mM, NaCl 200 mM pH 7.4 and 0.2 mM β-mercaptoethanol, at 3^o^C·min^-1^.

| **Enzymatic species** | **Protein concentration (μM)** | **T_m_**  **(^o^C) ^(1)^** | **ΔH**  **(kcal·mol^-1^)^(1)^** | **E_a_**  **(kcal·mol^-1^) ^(1)^** |
| --- | --- | --- | --- | --- |
| **D_Ma_-FlAsH** | 5 | 77.4±0.1 | 213±23 | 61±4 |
|  | 11 | 77.2±0.1 |  |  |
| **M_Ma_-FlAsH** | 5 | 53.6±0.1 | 67±9 | 48±6 |
|  | 11 | 54.0±0.1 |  |  |

^(1)^ Average±s.d. from at least four experiments carried out at different scan rates and/or protein concentrations.

**Table S3. Thermodynamic binding parameters for the interaction of Pex5p-pbd with AGT enzymes obtained by ITC analyses.**

| **Enzymatic species** | **Conditions** | **K_d_ (μM)** | **ΔH (kcal·mol^-1^)** |
| --- | --- | --- | --- |
| **D_Ma_-FlAsH** | Na-HEPES 20 mM, NaCl 200 mM, 0.2 mM ß-mercaptoethanol, pH 7.4, 10^o^C | 12±3 | 3.2±0.3 |
|  | K-phosphate 0.1 M, 0.2 mM ß-mercaptoethanol, pH 7.4, 10^o^C | 13±1 | 6.2±0.2 |
| **M_Ma_-FlAsH** | Na-HEPES 20 mM, NaCl 200 mM, 0.2 mM ß-mercaptoethanol, pH 7.4, 10^o^C | 25±5 | 5.4±0.4 |
|  | K-phosphate 0.1 M, 0.2 mM ß-mercaptoethanol, pH 7.4, 10^o^C | 28±4 | 6.3±0.6 |

**References**

1. Fargue, S.; Lewin, J.; Rumsby, G.; Danpure, C.J. Four of the most common mutations in primary hyperoxaluria type 1 unmask the cryptic mitochondrial targeting sequence of alanine:glyoxylate aminotransferase encoded by the polymorphic minor allele. *The Journal of biological chemistry* **2013**, *288*, 2475-2484, doi:10.1074/jbc.M112.432617.

2. Montioli, R.; Fargue, S.; Lewin, J.; Zamparelli, C.; Danpure, C.J.; Borri Voltattorni, C.; Cellini, B. The N-terminal extension is essential for the formation of the active dimeric structure of liver peroxisomal alanine:glyoxylate aminotransferase. *The international journal of biochemistry & cell biology* **2012**, *44*, 536-546, doi:10.1016/j.biocel.2011.12.007.
